# Association of Blood Lead (Pb) and Plasma Homocysteine: A Cross Sectional Survey in Karachi, Pakistan

**DOI:** 10.1371/journal.pone.0011706

**Published:** 2010-07-21

**Authors:** Mohsin Yakub, Mohammad Perwaiz Iqbal

**Affiliations:** Department of Biological and Biomedical Sciences, Aga Khan University, Karachi, Pakistan; Universidad Europea de Madrid, Spain

## Abstract

**Background:**

High blood lead (Pb) and hyperhomocysteinemia have been found to be associated with cardiovascular disease (CVD). Mean blood Pb and mean plasma homocysteine levels have been reported to be high in Pakistani population. The objective of the present study was to assess the relationship of blood Pb to the risk of hyperhomocysteinemia in a low income urban population of Karachi, Pakistan.

**Methodology/Principal Findings:**

In a cross sectional survey, 872 healthy adults (355 males, 517 females; age 18–60 years) were recruited from a low income urban population of Karachi. Fasting venous blood was obtained and assessed for blood Pb and plasma/serum homocysteine, folate, pyridoxal phosphate (PLP, a coenzymic form of vitamin B6) and vitamin B12. The study population had median (IQR) blood Pb of 10.82 µg/dL (8.29–13.60). Prevalence of high blood Pb (levels >10 µg/dL) was higher in males compared to females (62.5% males vs 56% females; p value = 0.05). Mean ± SD/median (IQR) value of plasma homocysteine was significantly higher in the highest quartile of blood Pb compared to the lowest quartile 16.13±11.2 µmol/L vs 13.28±9.7µmol/L/13.15 (10.33–17.81) µmol/L vs 11.09 (8.65 14.31) µmol/L (p value<0.001). Daily consumption of fruit juice had a positive influence on both levels of plasma homocysteine and blood Pb. Compared with the lowest quartile of blood Pb, the OR for hyperhomocysteinemia was 1.69 (95% CI, 1.00 to 2.85) for the fourth quartile when the model was adjusted for age, gender, folate and vitamin B12.

**Conclusions/Significance:**

This study showed a relationship between blood Pb and hyperhomocysteinemia in a general population of Karachi, Pakistan. The harmful effect of Pb on cardiovascular system could be due to its association with hyperhomocysteinemia.

## Introduction

Lead (Pb) accumulates in blood and other tissues when one gets exposed to it. There are no safe levels of lead within the body and the threshold for Pb levels in the blood has been reviewed over the past several years. On the basis of different epidemiological studies, it has been recommended that levels of blood Pb should be kept below 10 µg/dL [1], [2]. However, it has been reported that blood Pb level of even 2 µg/dL are associated with high rates of cardiovascular disease (CVD) and Pb level of 3.6 µg/dL could be responsible for 89% increase in mortality from cardiac disease [1]. The mechanism behind this deleterious effect of Pb is not clear; however blood Pb levels greater than 10 µg/dL have been considered to be responsible for increased rate of arteriosclerosis [3]. Hyperhomocysteinemia (levels of plasma homocysteine >15 µmol/L) has been shown to be associated with development of atherosclerosis and greater risk for CVD. Several studies have reported high levels of plasma homocysteine in Pakistani healthy adults and patients with coronary artery disease (CAD), [4]–[7]. Studies carried out in Pakistan show that environmental Pb pollution is very high in this country and the mean blood Pb concentrations in children and adults are well above the acceptable levels [8], [9]. In the Baltimore Memory Study, Schafer et al. [10], reported linear association between blood Pb and homocysteine in older adults. In another study carried out in two Asian countries, a clear relationship between blood Pb and homocysteine was indicated among individuals working in Pb stabilizer factory. This shows that there is a relationship between blood Pb and hyperhomocysteinemia [11].

Karachi is among the most densely populated industrial cities in South Asia. Because of a lack of clear cut policy by the Government of Pakistan regarding pollution control, heavy metal pollution contributed primarily by industrial waste is expected to be very high in large cities. Levels of Pb in ambient air of 3 major cities of Pakistan have been reported to be alarmingly high. The Pb contents in suspended particulate matter have been found to be in the range 1.03–4.8 µg/m^3^, while WHO/USAEPA guidelines suggest that Pb contents in ambient air should be 0.5–1.0 µg/m^3^ [12]. This is suggestive that urban population in a large city, such as Karachi would be at a greater risk of developing Pb toxicity. Therefore, exposure to Pb is a significant public health problem in Pakistan.

Keeping in view high prevalence of hyperhomocysteinemia and Pb pollution in Pakistani population it would be imperative to examine the relationship of homocysteine and blood Pb at an urban community level.

The aim of the present study was to investigate the relationship of blood Pb to the risk of hyperhomocysteinemia in a low income urban population of Karachi, Pakistan.

## Methods

### Ethics statement

The study was approved by the Ethics Review Committee of the Aga Khan University, and prior written informed consent was obtained from all the participants included in this study.

### Participants' enrollment

Eight hundred and seventy two healthy individuals (355 males and 517 females) of age 18–60 years from low income urban locality in east of Karachi, Pakistan were included in this study. In this cross sectional survey, a systematic random sampling was adopted to select houses. A sampling frame of 4000 houses was available in this locality through the Urban Health Project of the Department of Community Health Sciences, Aga Khan University for addressing the health and developmental needs of this community. The first house was selected randomly by computer draw, while every fourth household was selected for the sample applying the right hand rule for the direction. The selection of subject from each household was voluntary provided the participants fulfilled the inclusion criteria.

All the subjects were assessed for socio-demographic characteristics. Anthropometric measurements were also recorded by trained research assistants. They were community health workers from the same locality and were trained to collect demographic and clinical information from participants. Since juice consumption has been known to influence blood Pb levels [13], information on the number of times a person consumed fruit juices was also recorded. Data were collected as number of times juice consumed per month, per week or per day. The frequencies were then standardized to consumption per day.

Ten ml of fasting venous blood was collected and transferred equally into a heparinized tube and a non-heparinized tube. Whole blood was used for the determination of blood Pb, while plasma was analyzed for homocysteine and pyridoxal phosphate (PLP, a coenzymic form of vitamin B6). Serum was utilized for the determination of folate and vitamin B12 using radioassay [14], [15]. Plasma PLP was chosen as standard for vitamin B6 status and determined by modification of a method reported by Camp et al. [16], [17]. Determination of homocysteine was carried out by using a kit based on immunoassay (Abbott Laboratories, Ltd., Pakistan). Whole blood Pb was determined on graphite furnace using Hitachi Z-8000 Atomic Absorption Spectrophotometer with Zeeman's background correction, at the Pakistan Council for Scientific and Industrial Research Laboratory, Karachi. The minimum levels of detection in serum/plasma for folate, B12, PLP, homocysteine and the whole blood Pb were 0.5 ng/mL, 50 pg/mL, 3.2 nmol/L, 4 µmol/L and 1 µg/dL, respectively.

### Statistical Analysis

Continuous variables including blood Pb, homocysteine, folate, PLP, vitamin B12 and body mass index (BMI) were expressed as mean ± SD/median (IQR). All statistical analyses were done with the help of Statistical Package for Social Sciences® (SPSS) software version 13 for windows®. Independent sample t-test was used to compare mean values of continuous variables (blood Pb, homocysteine, folate, vitamin B12 and PLP) between two groups. Analysis of variance (ANOVA) was used to compare the above mentioned continuous variables across the blood Pb quartiles, followed by post hoc power estimation and Bonferroni test for multiple pair-wise comparisons.

Multiple linear regression was used to examine the relationship of log transformed blood Pb with log homocysteine levels, controlling for covariates (gender, occupation, ethnicity, education, BMI, and daily juice consumption). After that, known determinants of homocysteine (log folate, log PLP, and log B12) were added in the model. All those variables which were associated with homocysteine or influenced the relationship of log blood Pb with log homocysteine (p value based on change in F-statistic) were kept in the final analyses. Binary logistic regression analysis was also used to examine the association between risk of hyperhomocysteinemia (dependent variable) and blood Pb (independent variable). This model was also adjusted for age, gender, folate and vitamin B12 to assess the risk of hyperhomocysteinemia.

## Results

Study participants include 59.2% females and 40.7% males and had mean ± SD/median (IQR) age of, 32.51±10.71/30 (24–40) years. Analysis of the data revealed that mean ± SD blood Pb concentration 11.65±5.5 µg/dL in the study population was higher than the previously considered acceptable levels (<10 µg/dL) [18]. Approximately 59% of the study population had blood Pb levels greater than these levels. Moreover, the mean blood Pb concentration among males was significantly higher than females (p value <0.001). Blood Pb was also higher in subjects who are students or working in open environment compared to house wives/unemployed individuals (males and females); (p value <0.001) using Bonferroni test; Table 1.

**Table 1:** Demographic characteristics versus mean/median blood Pb levels in Pakistani adults.

Characteristics*	Blood Pb (*n* = 872)	p value**
**Age**		0.202
*≤20 years*	12.2 ( 4.6)/11.6 (8.4–15.0)	
*>20 years*	11.5 (5.6)/10.7 (8.3–13.5)	
**Gender**		<0.001
*Males*	12.6 (5.9)/11.3 (8.9–14.0)	
*Females*	11.0 (5.0)/10.5 (7.8–13.0)	
**Occupation**		<0.001
*Housewives/Unemployed individuals (males & females)*	10.9 (5.0)/10.5 (7.8–13.0)	
*Closed environment*	11.3 (4.1)/10.7 (8.5–13.0)	
*Students*	12.3 (4.0)/11.6 (8.9–14.2)	
*Open environment*	13.0 (6.8)/11.4 (9.0–14.8)	
**Ethnicity**		0.09
*Hindko/Hazara/Muree*	12.0 (6.0)/11.0 (8.6–14.0)	
*Pathan/Sawati*	11.1 (4.7)/10.4 (8.0–13.3)	
*Others*	11.4 (4.3)/10.5 (8.4–10.5)	
**Education**		0.04
*No education*	11.4 (4.8)/10.9 (8.3–13.5)	
*Middle (8^th^ grade)*	11.8 (5.3)/10.8 (8.2–14.0)	
*10^th^ grade*	12.4 (7.2)/11.1 (8.3–13.8)	
*≥12^th^ grade*	10.8 (3.8)/10.2 (8.1–12.9)	

*Values are expressed as mean (SD)/median (IQR).

**p value obtained using Independent sample t test for age and gender or from analysis of variance (ANOVA) for occupation, ethnicity, and education.

Table 2 shows the serum/plasma concentrations of homocysteine, folate, PLP and vitamin B12 and BMI by quartiles of blood Pb. Mean values of plasma homocysteine in blood Pb quartile 1 and quartile 4 were found to be significantly different (p value = 0.017) using Bonferroni pair-wise test. Moreover, post hoc power was determined and was still found to be more than 80%. No significant differences were observed among quartiles of blood Pb with respect to folate, PLP and vitamin B12. An inverse relationship between quartiles of blood Pb and BMI was observed (p value = 0.001).

**Table 2:** Serum/plasma concentrations of homocysteine, folate, PLP, vitamin B12 and BMI, by quartiles of blood Pb in Pakistani adults.

	Blood Pb				
Characteristics*	Q1 (*n* = 218)	Q2 (*n* = 218)	Q3 (*n* = 218)	Q4 (*n* = 218)	p value**
**Homocysteine, **µ**mol/L**	13.3 (9.7)/11.1(8.6–14.3)	14.6 (7.5)/12.7 (9.9–16.7)	15.2 (9.1)/12.5 (9.9–17.0)	16.1 (11.2)/13.1 (10.3–17.8)	0.017
**Folate, ng/mL**	6.9 (4.8)/5.6 (3.3–9.1)	6.8 (4.4)/5.9 (3.7–8.8)	6.3 (4.4)/5.0 (3.2–8.0)	6.4 (4.3)/5.3 (3.3–8.6)	0.3
**PLP, nmol/L**	34.9 (33)/28.6 (16.4–41.0)	32.2 (30.6)/25.1 (18.0–37.1)	31.9 (29.9)/26.5 (18.0–35.6)	33.4 (36.5)/25.6 (17.4–35.0)	0.7
**Vitamin-B12, pg/mL**	442 (230)/397 (300–532)	424 (223)/400 (270–540)	434 (232)/385 (280–532)	462 (231)/430 (313–565)	0.3
**BMI, kg/m^2^**	25.6 (5.8)/25.3 (21.1–29.1)	24.6 (5.3)/23.9 (20.5–28.1)	24 (5.5)/22.8 (20.0–28.1)	23.4 (5.9)/22.5 (19.1–26.8)	0.001

*Values are expressed as mean (SD)/median (IQR).

**p value is obtained by comparing mean (SD) values in four blood Pb quartiles by using one way ANOVA followed by Bonferroni test.

BMI = body mass index.

PLP = pyridoxal phosphate.

Table 3 shows the comparison of concentrations of plasma homocysteine, serum folate, plasma PLP, serum B12 and blood Pb in quartile 1 and quartile 4 on the basis of frequency of daily juice consumption. Mean plasma homocysteine and blood Pb levels in quartile 1 were found to be significantly higher than levels in quartile 4 of daily juice consumption (p value = 0.01 and p value <0.001, respectively). This indicates that increased daily consumption of fruit juice has a positive influence on both levels of plasma homocysteine and blood Pb. No significant differences were observed in the mean values of serum/plasma folate, PLP, and vitamin B12 for the lowest quartile of daily juice consumption compared to the values in the highest quartile.

**Table 3:** Concentrations of plasma/serum homocysteine, folate, PLP, vitamin B12 and blood Pb in quartile 1 versus quartile 4 of daily juice consumption.

	Daily Juice Consumption		
Characteristics*	Q1 (*n* = 453)	Q4 (*n* = 132)	p value**
**Homocysteine, **µ**mol/L**	15.3 (10.5)/12.5 (9.4–17.1)	13 (6.1)/11.7 (9.5–15.0)	0.01
**Folate, ng/mL**	6.7 (4.7)/5.6 (3.4–8.5)	6.9 (4.5)/5.6 (3.5–8.7)	0.7
**PLP, nmol/L**	32.6 (34.8)/25 (17–35)	39 (39.4)/30.6 (18.2–46.7)	0.07
**Vitamin-B12, pg/mL**	446 (238)/400 (280–560)	458 (248)/400 (294–547)	0.6
**Blood Pb, **µ**g/dL**	12.6 (6.6)/11.4 (8.8–14.7)	9.6 (3.5)/9.85 (7.4–11.4)	<0.001

*Values are expressed as mean (SD)/median (IQR).

**p value compares mean values of lowest quartile of intake (Q1) vs highest quartile of intake (Q4) using Independent sample t-test.

Pb = lead.

PLP = pyridoxal phosphate.

In order to evaluate predictors of log homocysteine adjusting for covariates, multiple linear regression was used. It turns out that an increase of 1 µg/dL log blood Pb was associated with an increase of 0.09 µmol/L log homocysteine. As homocysteine, in general, increases with age, separate models were repeated next in subjects' ≤20 years and >20 years. The association was stronger in subjects with age >20 years, such that an increase of 1 µg/dL log blood lead was associated with 0.1 µmol/L increase in log homocysteine compared to 0.07 µmol/L increase in log homocysteine in subjects ≤20 years. Moreover, this relationship remained significant for blood Pb, serum folate, serum B12, gender, daily juice consumption and occupation in subjects >20 years of age as compared to the subjects of age ≤20 years (Table 4). In unadjusted correlation analysis, log blood Pb was found to be modestly associated with log homocysteine (Pearsons's r = 0.18, p value <0.001).

**Table 4:** Predictors* of log homocysteine levels in Pakistani adults.

	Total (*n* = 872)		Age ≤20 years (*n* = 129)		Age >20 years (*n* = 743)	
	β(SE β)	p value	β(SE β)	p value	β(SE β)	p value
**Blood Pb** **	0.09(0.03)	<0.001	0.07(0.11)	0.115	0.10(0.03)	<0.001
**Folate** **	−0.18(0.02)	<0.001	−0.13(0.06)	0.005	−0.18(0.02)	<0.001
**PLP** **	−0.02(0.02)	0.07	0.04(0.06)	0.22	−0.03(0.02)	0.16
**B12** **	−0.16(0.02)	<0.001	−0.16(0.08)	0.15	−0.16(0.02)	<0.001
**Females**	−0.39(0.07)	<0.001	−0.36(0.16)	<0.001	−0.39(0.08)	<0.001
**Daily juice consumption**	−0.25(0.09)	0.01	−0.41(0.34)	0.23	−0.21(0.10)	0.04
**Occupational Exposure** ***		0.018		0.91		0.001
*Closed Environment*	0.05(0.07)		0.08(0.26)		0.05(0.08)	
*Open Environment*	0.04(0.07)		0.05(0.19)		0.04(0.08)	
*Students*	0.20(0.07)		0.15(0.17)		0.33(0.10)	

*Adjusted for variables in table as well as BMI.

**log transformed.

***Reference group are subjects who are housewives/unemployed (males and females).

Pb = lead.

PLP = pyridoxal phosphate.

Table 5 shows the odds of risk of hyperhomocysteinemia with quartiles of blood Pb. Compared to the lowest quartile of blood Pb (quartile 1), risk of hyperhomocysteinemia at different levels (quartiles 2,3 and 4) was found to be significant even when the model was adjusted for age, gender, folate and vitamin B12. However, no trend was observed in the odds of risk of hyperhomocysteinemia with quartiles of blood Pb.

**Table 5:** Risk of hyperhomocysteinemia with quartiles of blood Pb (95% CI).

	Q1	Q2	Q3	Q4
**Crude**	1	2.12 (1.38–3.25)	1.96 (1.27–3.01)	2.08 (1.35–3.19)
**Adjusted** *	1	1.89 (1.13–3.18)	2.21 (1.30–3.74)	1.69 (1.00–2.85)

*Adjusted for age, gender, folate, vitamin B12 levels and BMI.

## Discussion

Pb pollution is a serious environmental risk to human health, especially in South Asian countries where adequate regulatory measures are not in place. While studies have been carried out in developed countries to investigate the risk of CVD with mild hyperhomocysteinemia and blood Pb [1], [19], [20], there is hardly any report from the developing countries on relationship between plasma homocysteine and blood Pb at the community level. The present study provides information regarding increased risk of hyperhomocysteinemia when the blood Pb levels are high in a section of urban population of Karachi.

Our data also show significantly high percentage of men compared to women with blood Pb levels higher than the previously considered acceptable levels of blood Pb (<10 µg/dL; 62.5% vs 56%; p value = 0.05). These increased levels in men could be due to their greater exposure to environmental Pb pollution as they are generally the bread earners in this part of the world. Moreover, occupational exposure is an additive risk. Similar findings have been reported by Son et al. [21] who have shown relatively high concentration of blood Pb in Korean males compared to females. Students and persons working in an open environment with greater exposure to environmental pollution such as, laborers, vendors, bus conductors, vehicle drivers, construction workers had significantly higher levels of blood Pb compared to housewives and unemployed individuals (males and females); (p value <0.001). This indicates that greater exposure to environmental pollution could be one of the major determinants of high blood Pb in our urban population.

Increased mortality from cardiac events in individuals with blood Pb levels greater than 3 µg/dL [1] indicates that no levels of blood Pb could be considered safe. Thus, high blood Pb levels found in Pakistani population are suggestive that more efforts are needed to control Pb pollution in this country to bring down the high prevalence of CVD in this part of the world.

The observed linear association of plasma homocysteine with quartiles of blood Pb indicates that blood Pb could be another determinant of hyperhomocysteinemia. However, the known determinants of hyperhomocysteinemia, such as deficiencies of folate, vitamin B6 and vitamin B12 were not found to be associated with quartiles of blood Pb in the study population. It is unclear at this stage whether hyperhomocysteinemia is the cause or effect of high blood Pb. Some of the enzymes involved in homocysteine metabolism could be affected by Pb, thereby leading to hyperhomocysteinemia. The other possibility is that high levels of plasma homocysteine, a thiol amino acid with structure similar to dimercaptosuccinic acid could be chelating Pb, thereby delaying its plasma clearance [10]. The association between mean BMI and blood Pb found in this study has also been reported by others [10], [22].

Inverse relationship between daily juice consumption and mean levels of plasma homocysteine and blood Pb points towards the notion that adequate consumption of fruit and vegetable juices could be beneficial in reducing the risk of hyperhomocysteinemia and high blood Pb. In South Asian populations where mean levels of plasma homocysteine and blood Pb are quite high, public awareness about this simple way of bringing their levels down would have a positive influence on the health of masses in this region. Similar inverse relationship between fruit and vegetable juice consumption and plasma homocysteine has been demonstrated in men by Samman et al. [23] in Australian men. However, in their study, there was a significant increase in serum folate levels. Since in the present study no significant increase has been observed in serum/plasma levels of folate and PLP between quartiles of low and high daily juice consumption, it appears that decrease in homocysteine level because of increased fruit juice consumption could be due to a different mechanism. Studies have shown that high doses of vitamin C decrease levels of blood Pb probably through chelation [24], [25]. If a good proportion of blood Pb is bound to homocysteine, it is possible that its chelation by vitamin C present in fruit juice could enhance its excretion. Increased consumption of fruit and vegetable juices would have an additional benefit. These would also provide essential micronutrients in addition to vitamin C. Therefore, it would be a natural and healthy way of reducing the risk of hyperhomocysteinemia and high blood Pb. Lower levels of plasma homocysteine reduce the risk of development of CVD. Further studies would be needed to unravel the underlying mechanism.

In a previous communication, we reported mild association (Pearson's r = 0.24) between blood Pb and homocysteine [26]. However, in the present study, blood Pb was weakly correlated with plasma log homocysteine (Pearson's r = 0.18, p value <0.001).

The results from this study need to be evaluated within the context of its limitations. A major limitation was the dependence on a single blood Pb measurement which, perhaps, reflects recent exposure with half life of approximately 30 days for blood Pb [1]. Pb measurements, such as bone Pb and hair Pb would have been useful for more accurate depiction of Pb contents in the body that had accumulated over time showing long-term exposure. Analysis of vitamin C levels would have been helpful for the observed relationship between daily fruit juice consumption and blood Pb. Determination of the activity of enzymes involved in homocysteine metabolism in white blood cells would have been of help in ascertaining whether deactivation of these enzymes by Pb could be a cause of hyperhomocysteinemia.

Regardless of these limitations, the study did show a few novel findings. The relatively large sample size allowed analysis of mean homocysteine concentration in quartiles of blood Pb. The results are representative of an urban population of Karachi, Pakistan. High levels of blood Pb in men point towards the fact that Pakistani males are at a greater risk of developing hyperhomocysteinemia compared to females probably due to greater exposure to Pb pollution hence are more prone to developing CVD and cognitive disorders. Inverse association between daily juice consumption and plasma homocysteine concentration implies that increased intake of fruit juices could play a role in reducing hyperhomocysteinemia in Pakistani population. Moreover, the inverse relationship between juice consumption and blood Pb indicates that frequent consumption of juices could be an effective way of keeping the blood Pb levels low, thereby avoiding its deleterious effects. Investigation of any relationship between blood Pb and plasma PLP was another novel aspect of this study; however we did not find any association between these two parameters. Stronger association between log homocysteine and log transformed blood Pb in adults of age >20 years compared to adults ≤20 years indicates that older adults are at a greater risk of having elevated levels of homocysteine and blood Pb.

Further studies, especially on people with greater exposure to Pb would be helpful in ascertaining the relationship between blood Pb and plasma levels of homocysteine in such high risk group individuals.

Since hyperhomocysteinemia is now recognized as one of the major risks for the development of CVD, various factors associated with high levels of plasma homocysteine require immediate attention in populations having high prevalence of vascular disease. In the present study the focus was on high levels of blood Pb due mainly to rampant environmental pollution in an industrial city of this country.

This study showed a relationship between blood Pb and hyperhomocysteinemia in a general population of Karachi, Pakistan. Therefore, control of Pb pollution could be one of the important steps in reducing the burden of CVD.
